# Perilymph Fistula: Fifty Years of Controversy

**DOI:** 10.5402/2012/281248

**Published:** 2012-07-31

**Authors:** Jeremy Hornibrook

**Affiliations:** Department of Otolaryngology, Head and Neck Surgery, Christchurch Hospital, 2 Riccarton Avenue, Christchurch 8011, New Zealand

## Abstract

Perilymph fistula (PLF) is defined as a leak of perilymph at the oval or round window. It excludes other conditions with “fistula” tests due to a dehiscent semi circular canal from cholesteatoma and the superior canal dehiscence syndrome. It was first recognized in the early days of stapedectomy as causing disequilibrium and balance problems before sealing of the stapedectomy with natural tissue became routine. It then became apparent that head trauma and barotraumatic trauma from flying or diving could be a cause of PLF. Descriptions of “spontaneous” PLF with no trauma history followed. A large literature on PLF from all causes accumulated. It became an almost emotional issue in Otolaryngology with “believers” and “nonbelievers.” The main criticisms are a lack of reliable symptoms and diagnostic tests and operative traps in reliably distinguishing a perilymph leak from local anaesthetic. There are extensive reviews on the whole topic, invariably conveying the authors' own experiences and their confirmed views on various aspects. However, a close examination reveals a disparity of definitions and assumptions on symptoms, particularly, vestibular. This is an intentionally provocative paper with suggestions on where some progress might be made.

## 1. Introduction

Perilymph fistula (PLF) has been a controversial issue in otolaryngology now for fifty years. Many hold strong views on its existence or otherwise, the symptoms it might cause, the tests which might predict it, the reliability of what is described on exploration, and the effect of repair on symptoms. There are already excellent large reviews on the topic [1–3]. Here, key controversial aspects are discussed with the exception of surgical repair techniques. Suggestions for progress are offered.

## 2. Origin of the PLF Concept

Historically the term “perilymph fistula” has had a range of definitions. Gelle discovered that in Meniere's patients manipulation of the stapes could cause vertigo. In 1905, Hennebert [4] described nystagmus induced by alternating positive and negative pressure in the ear canal in syphilitic patients. “Hennebert's sign” came to be considered as diagnostic test for otological syphylis. Barany believed these observations were explained by hypermobility of the stapes. Ruttin [5] showed that Hennebert's sign was sometimes associated with a destructive lesion of the labyrinthine capsule by cholesteatoma. To this day, Hennebert's test is called a “fistula” test, but its features are now well explained by the stimulation of the ampullary receptor of one of the semicircular canals, usually the horizontal canal. The recent discovery of the superior canal dehiscence syndrome [6] makes it possible that, historically, some patients with an unexpected positive “fistula” test had that condition.

In the early days of stapes mobilization and stapedectomy surgery, in 1962, Farrior [7] reported that a polyethylene strut on a mobilized footplate had entered the inner ear so that perilymph escaped through the strut. Steffan et al. [8] added further examples of the “Slipped Strut Problem.” The potential for an oval window fistula was highlighted by Harrison et al. [9] who found forty-six fistulas, usually where gelatine sponge had been used as an oval window seal. The symptoms were vertigo, tinnitus, hearing fluctuation, imbalance, and aural fullness. They commented that the diagnosis is difficult because of the similarity of these symptoms to those of endolymphatic hydrops, a commonly repeated notion that persists to this day. In a later report on the long-term outcome of 1,943 stapedectomy operations Shea [10] could claim that the “The long debate about the value of Gelfoam as an oval window seal has just about ended” due to the use of a small prosthesis and natural tissue seal.

In 1968, Fee [11] noted that despite many reports of post-stapedetomy PLFs “it does not appear to have been recognized that fistulas can and do appear in normal ears after head injury.” This first report of traumatic PLF describes three patients with an oval window fistula following mild head injury or series of head injuries without concussion. The predominant symptom was “dizziness.”

In 1970, Stroud and Calcettera [12] introduced the term “spontaneous perilymph fistula”, based on four patients. A seven-year-old girl, one year after a modified radical mastoidectomy, was laughing and felt “something pop” in the ear accompanied by hearing loss, tinnitus, and nausea. A fistula at the stapes footplate was found and repaired, with later improvement in hearing. Three were adults (oval window PLFs) who all experienced vertigo and/or disequilibrium. In one symptoms began as she leaned over to wash her car. One was singing in a choir. One, in whom a loud noise such as gun fire could induce dizziness, attributed the onset of his symptoms to when he was boxing in the army. Therefore, one was postsurgical and one had an identifiable trauma history.

## 3. Institutional Series

The recognition that PLF could occur without stapes surgery or trauma initiated widespread interest, resulting in numerous institutional reports on their experience and results, particularly in the United States, Seltzer and McCabe [13]. Reported on the Iowa experience on PLF in a hundred and seventy-seven patients. The most common symptom presentation was a combination of hearing loss, tinnitus, and vestibular symptoms which were predominantly disequilibrium and motion intolerance. Other than post-stapedectomy the commonest cause was trauma (direct, barotrauma, acoustic), and straining. In 24% there was no identifiable cause.

The Stanford experience of PLF over 11 years was reviewed by Shelton and Simmons [14]. Seventy-eight ears were explored for reasons which varied over the period. No specific diagnostic tests were attempted. A PLF was diagnosed in 50%, but the oval and round windows were all grafted whether there was a fistula or not. The most common symptoms were vestibular described as “postural unsteadiness” but some were said to have vertigo. Of the patients were no fistula was found 44% reported improvement or cure of their symptoms. 51% had no identifiable cause and were called “spontaneous.”

 In the Dartmouth-Hitchcock Medical Centre New Hampshire Experience by Weider and Johnson [15], thirty-five fistulas were diagnosed in thirty-nine ears of thirty-five patients. In 79% of the patients with “spontaneous PLFs” the symptoms began soon after an event involving physical or mechanical stress.

 The House Ear Clinic experience was reviewed by Rizer and House [16]. Over a twelve year period the ears of eighty-six patients were explored. A preoperative “clinical fistula test” was listed but not described. 41% had a fistula. The main symptoms were “dizziness” and hearing loss, but not tinnitus. In contrast where tinnitus was the chief symptom no fistula was found. Very few had an improvement in hearing. When a fistula was found 68% had an improvement in their major symptom, but when a fistula was not found 29% felt better, suggesting a placebo effect. In those where a fistula was found one third had no history of ear surgery or trauma. On the basis of their results, the House Ear Clinic advocated a very cautious approach to the diagnosis of a PLF, especially for sudden hearing loss and in children [17].

Meyerhoff and Pollock [18] from the University of Texas Southwestern Medical Centre explored the ears of a hundred and twenty patients with a variety of symptoms: balance disturbance with or without progressive hearing loss and with or without a traumatic event, sudden sensorineural hearing loss, and also tinnitus as sole symptom. Both windows were patched regardless of the findings. The greatest improvement in symptoms was in the balance disturbance group with a trauma history, and the worst in those with tinnitus or sudden hearing loss only.

In the Portland Experience on the surgical management of perilymph fistulas Black and colleagues [19, 20] found seventy-nine fistulas in ninety ears (88%). 52% were oval window, 20% were round window, and 22% were both. A trauma history was elicited in nearly all. The main symptom was “disequilibrium” (90%) with subjective and objective aural symptoms being half a common. “Cognitive dysfunction” was also a feature.

## 4. Mechanisms of PLF

In a 1971 presidential address to the Triological Society Goodhill [21] advanced a theory of labyrinthine window ruptures as a possible cause of sudden deafness associated with exertion or trauma. This interest was stimulated by Stroud and Calceterra's [12] suggestion that increased perilymphatic pressure had caused a window “rupture” in their patients with a “spontaneous” PLF. The two proposed mechanisms were explosive and implosive. “Explosive” would require an increase in cerebrospinal (CSF) pressure, transmitted from the internal auditory meatus or by the cochlear aqueduct. The theory proposes that a force up an abnormally patent cochlear aqueduct could rupture the basilar membrane and Reisner's membrane into the scala vestibuli, and conceivably injure the utricle, saccule, the semicircular canal system, the round window membrane, or the annular ligament of the stapes. Conversely, an “implosive” force would be from a valsalva manoeuvre causing sudden air pressure increase through the Eustachian tube, a sharp increase in intratympanic pressure and rupture of the round window membrane or the annular ligament of the stapes. Goodhill proposed that a PLF could be a cause of sudden sensorineural hearing loss with and even without a history of trauma [22, 23]. His theory stimulated an interest in possible round window ruptures from either of these mechanisms. In 1975, Tonkin and Fagan [24] reported on thirteen patients with a round window fistula where the initiating event appeared to be direct head trauma in four, but exertion, barotrauma from flying and diving, acoustic trauma, vomiting, and postoperative in the remainder. They stressed that the incident could be long forgotten by the patient. At the Royal Ear Nose and Throat Hospital in London in a seven-year period, thirty-two patients had a confirmed PLF [25]. When the cause was blunt head trauma the fistula was always at the oval window. When the cause was barotrauma, exertion or unknown was always at the round window. Both these studies give some credence to a possible implosive/explosive mechanism for a round window fistula, but suggest that for an oval window fistula something else is required.

 Early temporal studies in the 1930s [26] showed that a crack between the round window niche and the ampulla of the posterior canal was not uncommon, but it was assumed to be an artefact. Subsequently, this has been shown to be developmental [27] and that similar microfissures superior and inferior to the oval window occur [28]. These findings were the impetus for Kohut's temporal bone studies on patients who *might* have had a PLF [29]. On the* assumption* that PLF patient ears would have endolymphatic hydrops, the paired temporal bones of eleven patients with histological hydrops and from eighteen patients with no vestibular symptoms (and normal hearing) were examined in regard to oval and round window features. In all the normal bones, the fissula ante fenestram was closed by cartilage or lamellar bone, and the round window niche fissure was sealed by collagen or bone. In the bones with hydrops one had a round patent round window fissure and a history of vertigo attacks which had been diagnosed as Meniere's disease. One had a “patent” fissula ante fenestram containing only fibrous tissue and a patent fissure between the round window niche and a history of “waxing and waning disequilibrium” that could have been due to a PLF. Earlier temporal bone studies showed that potential patency of the fissula is present at birth [30]. Kohut suggested that a “patent” fissula ante fenestram or round window fissure could be a predisposing congenital feature that could lead to a perilymph leak.

 There is an almost total absence of detailed cases of postmortem histology on ears with a premortem diagnosis of a treated PLF. The only one has been provided by Kohut et al. [31]. A sixty-eight-year-old man had a 43-year history of disequilibrium and hearing loss. This began at the age of twenty-five years with hearing loss in the right ear. At the age of 58, after being next to a five inch firing gun, his hearing in the right ear and balance became worse and sneezing would make him stagger. Exploration of the right ear revealed a PLF at the fissula ante fenestram and of the floor of the round window niche, and these were repaired with connective tissue. His balance returned, but not the hearing. The patient died four years later and his temporal bones were obtained for histology. On the left (unoperated) side the fissula antefenestrum and the round window fissure were not potentially patent. In the operated (right) ear the fissula antefenstrum and round window were “patent.” Of particular note there was no histological evidence of hydrops in either ear. Kohut states that this man's main symptom was “constant disequilibrium.”

## 5. Children

In Goodlhill's paper [22] on sudden sensorineural deafness diagnosed as having PLF two were children with a history of exertion. PLF in children causing hearing loss became a topic of interest in paediatric otolaryngology. Grundfast and Bluestone [32] described six children with rapid onset of sensorinerual hearing loss, disequilibrium, or both, with PLF, following exertion or otitis media. Later, at the same institution over a seven year period forty-four ears were explored for PLF which was found in 66% [33]. After the surgery, the hearing was unchanged in 86%, improved in 5%, and worsened in 9%, but the vestibular symptoms resolved in all in whom a PLF was repaired. Congenital middle ear abnormities were present in nearly 50% of ears. Congenital ear abnormalities likely to be associated with a PLF are an abnormal stapes, cochlea or vestibular dysplasia, and a dilated vestibular aqueduct [34]. In other institutional series on children with progressive sensorineural hearing loss the confirmation of a PLF occurred in only 11% of explored ears, even though half had a radiological inner ear abnormality [35]. At the Children's Hospital in Sydney, Australia, over an eight-year-period, forty-nine ears of children with fluctuating deafness, sudden hearing loss, and “vertigo” were explored. A PLF was diagnosed in forty (82%), with no improvement in hearing at six months and a later progressive hearing loss in both the operated and nonoperated ears [36]. Only ten had a definite congenital abnormality.

There is a wide disparity of confirmed PLFs and underlying causes in children whose ears are explored for the same reasons. However, there is agreement that when the predominant symptom is hearing loss recovery of hearing is rare.

## 6. “Spontaneous” PLF

In 1989, a questionnaire on PLF management was sent to members of the American Otological Society and the American Neurotological Society. The number of PLF explorations per year ranged from none to fifty, with median of five per year. 75% percent of respondents said they would graft a window even if a fistula was not found [37].

The term “spontaneous PLF” had been introduced by Stroud and Calcettera [12] and became increasingly used. A key event in the PLF debate occurred in 1992 when Shea [38], in a 1992 Otolaryngology-Head and Neck Surgery editorial entitled “The Myth of Spontaneous Perilymph Fistula,” wrote: “I do not believe there is such a thing as spontaneous perilymph fistula, or, if there is it is so rare that in doing more than 36,000 otologic operations during the last 39 years I have never recognized a patient with it…I believe the modern interest in spontaneous perilymph fistula began in the minds of a small group of “true believers”…no characteristic signs, symptoms or diagnostic tests exist for spontaneous perilymph fistula.”

 Shea's invective stimulated letters to the editor, some supporting and some countering that claim [39], stressing that most “spontaneous” PLFs are attributable to a traumatic event which the patient has forgotten. Cole [40] countered the claim in “The Validity of Spontaneous Fistula.” Over a three-year, period forty ears of forty patients were explored under local anaesthetic on an outpatient basis. Of the forty possible fistulas, twenty seven were “event-related” (77% positive) and thirteen “spontaneous” (76% positive). The commonest symptom combination was vestibular and auditory. The prior “events” were middle ear surgery, head injury, blast injury, ear slap, weight lifting and labor, and delivery. In both groups, symptoms had been present for a range of just days to twenty years. In the ninth with a “spontaneous” PLF, no cause was known or suggested, and both windows were grafted none the less.

There is a wide disparity in the rates of confirmation of “spontaneous” or idiopathic PLFs. Routine window patching when there is no fistula is common and seems certain to confound the interpretation of presenting symptoms, diagnostic tests and outcomes.

## 7. Trauma

 Trauma from head injury, flying and diving barotrauma, sneezing, coughing, and labor as the most common cause of a PLF has been a feature in all the institutional series discussed. Three novel causes have been lightening strike [41], airbag trauma [42], and acoustic trauma from a fire engine siren [43].

 Two papers have focussed on head trauma and whiplash as a cause of PLF. Fitzgerald [44] described five patients with a PLF caused by head injury, whiplash, and gunshot impact. Most had disequilibrium, nausea, and anxiety or cognitive problems assumed to be postconcussive syndrome.

Grimm and colleagues [45] performed detailed neurological and neurootological studies on one hundred and two adults with mild defined cranio-cervical trauma who had a confirmed PLF. The predominant symptom was “disequilibrium, dizziness,” motion intolerance, nausea and memory loss, stiff neck, and headache. Hearing loss was a less common feature. Grimm emphasised that these symptoms can be easily assumed to be postconcussion syndrome. He has suggested the subtle symptoms of a chronic PLF make it a neurological syndrome as well as otological.

After an inner ear injury, there is nearly always recovery or central adaption. However, a PLF is a rare example of an *unstable* peripheral organ. The vestibular system is a very primitive aspect of brain function which is preoccupied with calculating gravity and orientation to earth-vertical, so when it is perpetually confused higher brain function may become subtly involved [46].

## 8. Operative Confirmation of PLF

The volume of perilymph is estimated to be approximately 75 microlitres, so confirming a leak usually entails visualizing a tiny quantity of fluid, unless it is dramatic which probably means a CSF leak. Consequently, the volume of fluid collectable for a chemical test is miniscule.

Standard technique for visually confirming a fistula is prolonged examination with microscope and asking the anaesthetist to increase intrathoracic pressure. The exit of perilymph is rarely rapid, but episodic and often appearing a tiny bead with a changing light reflex, reappearing after it is suctioned. However, surety that it is not clear local anaesthetic fluid remains a problem.

In a chinchilla study [47], intravenous fluoresceine was found to enter perilymph rapidly and long before it reached the CSF, suggesting that perilymph is produced by the cochlea. The report that intravenous fluoresceine assisted the intraoperative detection of PLF [48] aroused the hope that this could be the ideal simple technique, soon dispelled by two animal studies [49, 50] showing fluorescence around the round and oval window niches from fluid transudates, with only weak or nonfluorescence of perilymph. Intrathecal administration of fluoresceine has been no more successful, has potential complications and has not been recommended [51].

A novel “reverse” use of fluorescine is its combination with injected local anaesthetic, helping to distinguish clear perilymph from green stained tissue and fluids at the round and oval windows [52].

The most commonly employed chemical test to distinguish perilymph from other fluids has been beta-transferrin, which is in perilymph and cerebrospinal fluid (CSF) but not plasma. In a prospective single-blinded trial, gelfoam was placed in the round and oval windows of patients with a suspected fistula and in patients having other otological procedures. Tests for beta-transferrin were positive in 66% of those with a fistula [53]. In another study [54] beta-transferrin was detected in only 5% of samples from twenty PLFs. The concentration of beta-transferrin in perilymph is only 50% of its concentration in CSF [55]. A typical clinical sample of PLF fluid is 0.5 microlitres and often contaminated with plasma or local anaesthetic.

Cochlin-tomoprotein (CTP) is a novel perilymph-specific protein, not found in CSF, saliva, or serum [56] and found in a perilymph leak in a patient with a penetrating ear drum injury [57] and in some cases of PLF [58].

 Poe et al. [59] used fiberoptic and rigid endoscopes to visually confirm surgically created window fistulas in five cats, and then the ears of twenty patients with suspected PLFs, without injected local anaesthetic. In the patients no fistula was seen. However, in a later study in which endoscopy was performed just prior to tympanometry in three patients. In all the endoscopy showed no fistula but at tympanotomy there was clear fluid emerging at one or both windows, reinforcing the point that clear fluid may not be perilymph [60]. Ogawa and colleagues [61] used a superfine flexible endoscope (through the Eustachian tube) and angled “needle” rigid scope in five patients with a suspected PLF and in normal volunteers. With the transtubal flexible scope an adequate view of the windows is rarely obtained. In three patients with a round window PLF the fistula was seen prior by rigid endoscopy. Karhuketo and Puhakka [62] have endoscopically repaired an round window fistula in a diver. Selmani and colleagues [63] endoscoped one ear of two hundred and sixty-five patients with Meniere's disease, recurrent vertigo, progressive hearing loss, sudden deafness, otosclerosis, and suspected PLF. In general the round window could nearly always be viewed by a 5 degree rigid scope and the fissula ante fenestram edge of the oval window by a 25 degree scope. Because only one PLF was seen they concluded that endoscopy may be of a limited value for diagnosing that condition.

 It seems that office endoscopy of the round and oval windows is usually achievable, but there are conflicting assumptions from what is observed.

 Intraoperative EcochG has been used for certain PLF diagnosis, discussed below.

## 9. Animal Studies and Electrocochleography

In animal models of PLF caused by removing or breaching the round window membrane in guinea pigs and cats [64–67] histology and auditory brainstem hearing thresholds suggest that PLFs can heal, that there may be no long-term hearing loss, and sometimes cochlear hydrops is observed.

Electrocochleography (EcochG) has been used both in animal histological studies, and as an office fistula test and intraoperatively in humans.

Meyerhoff and Yellin [68] performed EcochGs on thirty-nine ears before and after exploration for PLF. Of the twenty ears with an “abnormal” SP/AP ratio (>0.37) sixteen had a PLF compared with only one with a “normal” ratio. Postoperatively, in eighteen of the twenty positive ears, the SP/AP returned to “normal,” suggesting an inner ear disturbance and recovery.

Arenberg and colleagues [69] performed click stimulus EcochG on twenty-seven patients with a confirmed PLF. Based on an SP/AP ratio of >0.5 in fourteen the EcochG was “abnormal” and in some cases recovered post-op. In a guinea-pig animal model perilymph was allowed to exit “inactive” or suctioned from the window “active” made into the cochlea when the cochlear aqueduct was blocked and unblocked. There was a consistent increase in the SP when perilymph was suctioned. In seven of the twelve animals with “active” PLF later histology showed some evidence of hydrops, which is not seen in any control animal.

Gulya and colleagues [70] also performed click stimulus EcochG in guinea pigs before and after creation of a round window fistula with a hook, but without suction. No change had occurred in the post-op SP/AP ratio. An equal number of PLF animals and controls showed a subtle basal turn “hydrops.”

Gibson [71], a pioneer of electrocochleography, used intraoperative tone stimulus EcochG during stapedectomy and cochleostomy surgery with a silver ball electrode placed on the round window or oval window. After a stapedotomy there was no change in the EcochG unless perilymph was suctioned when there was dramatic decrease in the size of the AP and increase in the SP. The anaesthetist was asked to increase the intrathoracic pressure, resulting in recovery of the AP and decrease in the SP when perilymph refilled the otic capsule. This observation was developed into an office diagnostic test for PLF when the transtympanic needle is placed in the round window niche. After click stimulus baseline test another is done as the subject performs a closed glottis valsalva manoeuvre. A strongly positive test is when the click AP increases with the valsalva manoevre. Of two hundred and forty-six ears with possible posttraumatic PLF ninety had a positive test. Of those forty-six ears were surgically explored and a fistula diagnosis made in 88%.

Gibson has also used the intraoperative EcochG as a method of both proving and disproving the presence of a window fistula. In one hundred and twenty-three children with congenital hearing loss who were tested using transtympanic EcochG with a “golf club” electrode inserted through a posterior myringotomy no fistulas were identified, even in anatomically abnormal ears [72]. In two patients intraoperative EcochG with suction demonstrated a nonvisible window PLF, which the office valsalva test had predicted [73].

## 10. Vestibular and Balance Tests

A early attempt on the use of ENG testing for eliciting nystagmus by canal pressure with a pneumatic otoscope (Hennerbert's sign) predicted the presence of PLF in some patients [74]. This implies stimulation of the vestibulo-ocular reflex so that the stimulus was transmitted to the horizontal canal receptor and would presumably require a large defect. 

 Black and colleagues [75] used sinusoidal (300–500 mm H_2_O) ear canal pressure stimulation in patients with platform posturography to stimulate postural reflexes, reflected by postural sway. Perilymph fistulas were confirmed in 97% of the seventy-five ears that had a positive test.

In a multi-institutional partially blinded study, Sheppard and colleagues [76] used canal pressure and platform posturography to test patients with suspected PLF and other peripheral balance disorders. Six testing protocols were compared. Only some surgeons were aware of the test results. The overall conclusion was a 56% diagnostic specificity for a confirmed PLF.

In 1929, Tullio [77] showed that loud sounds could induce nystagmus in dogs with surgically fenestrated superior canals and head tilting and leg flexion in pigeons and rabbits with intact labyrinths. There has been a revival of interest in the Tullio phenomenon as a feature of the superior canal dehiscence syndrome [6], in which an abnormally reduced myogenic vestibular potential (VEMP) is now an essential aspect of the diagnosis [78].

The possible relevance of the Tullio phenomenon to PLF diagnosis has been considered [79–81]. Pyykko and colleagues [80] tested fifty-seven control subjects, seven with different inner ear pathologies, and seven with a suspected PLF on a force platform with a thirty second low frequency sound stimulation to the ear. All the PLF patients showed altered postural stability, but not the controls with a pure sensorineural hearing loss. About 20% of patients with other conditions (including Meniere's disease) showed an abnormal response. Tonkin and colleagues [81] also used a 250 Hz tone in standing patients and found a 77% specificity with a confirmed PLF. False positives may have been due to a startle reflex. Clearly it is not specific for PLF but it appears a logical investigation. As the Tullio phenomenon is stimulating a purely vestibulospinal response, it is initiated from the otolith organs.

 There are numerous descriptions of clinical balance testing on PLF patients, as variations on the Romberg test. The Fukuda/Unterberger test is well accepted as a clinical test for demonstrating postural turning or instability from vestibular *hyopfunction*. That PLF patients may have a *unique *balance problem was first suggested by Singleton [82]. For the “eyes-closed turning test” patients are asked with walk forward with eyes closed and then turn quickly with one step and stop. A positive test is the inability to stay stable. Twenty-three of twenty-six subjects with a fistula had a positive test which appeared negative in patients with other causes of dizziness. It is only a clinical balance test, but its use and objective verification merit some scrutiny.

## 11. Summary and Future Directions

 Criticisms of all aspects of a possible PLF (causes, likely symptoms, preoperative tests and observations at operation and outcomes) are all valid. It is seems well agreed that the (now unusual) main symptoms of a PLF following stapedectomy are vestibular, mainly “dizziness” or disequilibrium. There is evidence that barotrauma (diving, sneezing coughing, labor, and acoustic trauma) can initiate a PLF. There is poor evidence that PLF is a cause of sudden hearing loss, *unless *there has been a distinct prior traumatic event. There is good evidence that head trauma (even mild) and whiplash can initiate the onset of a PLF. Many authors emphasise that such likely event can be forgotten, or even concealed by the patient. Although the term “spontaneous” has been extensively used, a more appropriate term would be *idiopathic*. It appears that window repair for hearing loss or tinnitus from a suspected PLF from any cause rarely results in improvement. Barotraumatic “implosive” causes are most likely to cause a round window PLF, and head trauma are most likely to cause an oval window PLF. At the round window fistulas are sometimes described as a hole or tear in the round window membrane, but often as a smallbead of perilymph emerging from the round window fissure at its inferior edge. At the oval window a fistula is nearly always at its anterior edge, that is, at the fissula ante fenestram. This support's Kohut's view that a PLF, particularly from head trauma, is most likely to occur in individuals who have a congenital potential “patency” at those two sites [29]. The evidence for this came from temporal bone histology, which is time-intensive and expensive. The number of temporal bone laboratories rose from four in 1910 to thirty-two in 1984. Mainly because of financial constraints there are now only three [83]. 

Consequently temporal bone otopathology is now difficult to access. Advances in imaging are likely to take its place. The resolution of temporal bone imaging by computed tomography (CT) and by magnetic resonance imaging (MRI) is improving. In a cat model with surgically created round window PLFs intrathecal gadolinium was seen on MRI in the cochlea and ipsilateral mastoid bulla [84]. Using intravenous gadolinium in a six-year-old boy with a congenitally abnormal ear, high resolution T2-weighted MRI showed a fluid leak in the middle of the stapes footplate which was surgically confirmed [85]. There have been reports of intralabyrinthine air seen on CT in patients with round window fistulas [86, 87]. High resolution CT can now image normal and abnormal stapes in considerable detail [88] and has shown a subluxed stapes in an eleven-year-old boy from a traumatic penetrating injury [89]. As yet there are no reports of high resolution CT imaging of a “patent” or potentially “patent” fissula ante fenestram.

Certain proof of a fistula at exploration remains problematic. The new perilymph-specfic CTP [56–58] shows some promise but will require verification from other centres. As with any chemical test the result of the assay is unlikely to be available during the operation. In that regard a change on the intraoperative EcochG [71–73] is the most unequivocal test that a window fistula is present, but it requires special equipment and is unavailable to most. The addition of fluoresceine to local anaesthetic [52] to distinguish it from clear perilymph has been the most simple, low-cost and practical contribution to perilymph identification.

 A recurring claim is that individuals with a PLF have endolympahtic hydrops in the affected ear [9], and that this is the reason for their vestibular symptoms, requires some scrutiny. In early animal experiments using click stimulus EcochG the response did not change unless perilymph was suctioned, suggesting a change in inner ear dynamics. However, a normal click SP/AP ratio of >0.37 is not adequate to make an electrophysiological diagnosis of hydrops. In animal models of PLF some animals show histological evidence of hydrops, but this is not proof that hydrops is the cause of the predominant vestibular symptoms in humans. Tone burst EcochG is a far more sensitive test for the degree hydrops that would be expected in Meniere's disease, and there is a need for patients a proven PLF to have been tested by this and other techniques [90]. In most inner ears intratympanic gadolinium passes through the round (and/or oval) windows to distinguish perilymph from endolymph on MRI, thereby showing endolymphatic hydrops.

 What are the predominant vestibular symptoms of a PLF? The terms used for PLF vestibular symptoms have been “dizziness”, “imbalance”, “disequilibrium”, and often “vertigo”. In contrast to “dizziness” vertigo has always had at its most simplest level a well understood definition of an hallucination of motion, but in the PLF literature the word has been used loosely, and probably as a term to cover any vestibular symptom. If the PLF patient is described as truly experiencing vertigo it implies a discrete attack of rotational vertigo caused by Meniere's disease or something resembling it. If that is the case it should be *personally witnessed* (to confirm spontaneous nystagmus) by the clinician. With the best of intentions one cannot diagnose the cause of a patient's vestibular symptoms purely by their description of them. Similarly claims that PLF patients have positionally-induced nystagmus may be explained by coincidental benign positional vertigo.

The Barany Society has sought to refine the definition of common vestibular symptoms [91]. Vertigo is “the sensation of self-motion when no self-motion is occurring or the sensation of distorted self-motion during an otherwise normal head movement.” Dizziness is “the sensation of disturbed spatial orientation without a false or distorted sense of motion”.

PLF patients routinely do not describe either of these. The most predominant symptom is of being “off balance” or disequilibrium. This again raises the question of whether PLF patients have a *unique* balance abnormality that is not explained by hydrops or by vestibular *hypofunction* in the affected ear. References to the implication that the disequilibrium of PLF patients are attributable to a specific otolith organ dysfunction are rare [92], but it is a possibility which warrants further investigation. As yet there are no VEMP studies in PLF patients, but an abnormal VEMP in a PLF ear may provide (other than possible hydrops) some evidence. Claims for clinical balance tests demonstrating a specific PLF sign unrelated to inner ear *hypofunction * [82] require objective verification.

In summary, PLFs do occur, and usually there has been an identifiable traumatic event. Hearing improvement from a PLF repair (of any cause) is rare, but that is not a reason not to explore the ear if there is strong evidence and a likelihood of the hearing loss advancing. The true existence of fistulas and the outcomes of surgical repairs have been confounded by studies where window grafting has been done whatever has been observed. The most common symptoms of a PLF are vestibular, but a confusing range of unverified terms has been used and needs to be clarified. The possibility that PLF patients have a unique balance problem due to otolith organ dysfunction unrelated to hydrops merits further investigation. Eventually advances in imaging may “image” the fistula. Meanwhile, when a PLF is strongly suspected a simple tympanotomy is justified.
